# The Future of Orthodontics: Deep Learning Technologies

**DOI:** 10.7759/cureus.62045

**Published:** 2024-06-10

**Authors:** Aathira Surendran, Pallavi Daigavane, Sunita Shrivastav, Ranjit Kamble, Abhishek D Sanchla, Lovely Bharti, Mrudula Shinde

**Affiliations:** 1 Department of Orthodontics & Dentofacial Orthopaedics, Sharad Pawar Dental College & Hospital, Wardha, IND

**Keywords:** ai-assisted robotic orthodontics, 3d imaging, machine learning, deep learning, artificial intelligence

## Abstract

Deep learning has emerged as a revolutionary technical advancement in modern orthodontics, offering novel methods for diagnosis, treatment planning, and outcome prediction. Over the past 25 years, the field of dentistry has widely adopted information technology (IT), resulting in several benefits, including decreased expenses, increased efficiency, decreased need for human expertise, and reduced errors. The transition from preset rules to learning from real-world examples, particularly machine learning (ML) and artificial intelligence (AI), has greatly benefited the organization, analysis, and storage of medical data. Deep learning, a type of AI, enables robots to mimic human neural networks, allowing them to learn and make decisions independently without the need for explicit programming. Its ability to automate cephalometric analysis and enhance diagnosis through 3D imaging has revolutionized orthodontic operations. Deep learning models have the potential to significantly improve treatment outcomes and reduce human errors by accurately identifying anatomical characteristics on radiographs, thereby expediting analytical processes. Additionally, the use of 3D imaging technologies such as cone-beam computed tomography (CBCT) can facilitate precise treatment planning, allowing for comprehensive examinations of craniofacial architecture, tooth movements, and airway dimensions. In today's era of personalized medicine, deep learning's ability to customize treatments for individual patients has propelled the field of orthodontics forward tremendously. However, it is essential to address issues related to data privacy, model interpretability, and ethical considerations before orthodontic practices can use deep learning in an ethical and responsible manner. Modern orthodontics is evolving, thanks to the ability of deep learning to deliver more accurate, effective, and personalized orthodontic treatments, improving patient care as technology develops.

## Introduction and background

Over the last 25 years, the use of information technology (IT) in the field of dentistry has increased substantially. Artificial intelligence (AI) facilitates the examination, organization, visualization, and cataloging of medical data. For example, AI algorithms enable the evaluation of dental images and cone-beam computed tomography (CBCT) images, identifying cephalometric landmarks, performing cephalometric analysis, and predicting treatment outcomes. Its sophisticated pattern recognition and predictive algorithms are driving scientific discoveries across various disciplines. The current paradigm is machine learning (ML), a term coined by Arthur Samuel in 1952. ML and symbolic AI differ primarily in that, in ML, models learn from real-world examples instead of human-established rules [1].

Deep learning, a subtype of AI, enables computers to learn and make decisions without explicit programming. These algorithms, modeled after the neural networks of the human brain, have a wide range of uses in many industries, including medicine. In orthodontics, deep learning has become an invaluable technique for enhancing treatment planning, diagnosis, and outcome prediction. The integration of advanced technologies in orthodontics has revolutionized the design, implementation, and evaluation of treatment in the modern era [2, 3]. In the past, orthodontic specialists have been the primary authorities for diagnosing and addressing orthodontic issues through their analysis of radiographs, clinical findings, and patient documentation. However, deep learning algorithms can extract significant patterns and insights from large volumes of data, including medical records, cephalometric radiographs, and 3D imaging. This capability allows for more comprehensive and data-driven treatment planning, resulting in more precisely tailored and effective orthodontic therapies [4].

One common application of deep learning in contemporary orthodontics is the automation of cephalometric analysis. To reduce human error and streamline the analysis process, deep learning algorithms may be trained to recognize anatomical features on radiographs. These models can help in the early detection of orthodontic abnormalities, which can lead to better treatment outcomes and earlier intervention. CBCT, one of the 3D imaging technologies, has also enabled deep learning in orthodontics. Deep learning algorithms can analyze 3D imaging data to obtain valuable insights regarding tooth movements, airway dimensions, and craniofacial design. This facilitates proper treatment planning and contributes to a more complete image of the patient's oral and facial structure [5, 6]. In this era of personalized medicine, the use of deep learning in orthodontics offers the potential to tailor treatments to the specific needs of each patient. By using data analytics and ML, orthodontists can estimate treatment results, make well-informed decisions, and improve treatment plans for greater efficacy and efficiency.

## Review

History and evolution

Numerous studies have demonstrated that incorporating AI into orthodontic clinical practice can increase its effectiveness. Notably, several AI-powered software systems, including Mastro 3D V6.0 (ASTRON d.o.o., Slovenia), Uceph 4.2.1 (TOMA Dental, South Korea), and 3Shape Dental System 2.22.0.0 (3Shape A/S, Denmark), have been widely utilized in orthodontic therapy. Their use has resulted in significant improvements in the efficiency and accuracy of orthodontic treatment plans [2, 7]. With advancements in AI algorithms, processing capacity, and dataset accessibility, the use of AI in orthodontics is expanding and improving patient outcomes.

Researchers can gain a thorough understanding of the rapidly changing field of orthodontics by regularly reviewing summaries of the latest advancements in artificial intelligence applications. The use of AI in orthodontics has shown promising results, but there is still considerable room for improvement. This study will cover diagnosis, treatment planning, and clinical implementation to offer a comprehensive evaluation of the status of AI applications in orthodontics. In addition to addressing current limitations of AI, the study provides potential future directions and valuable insights for utilizing AI in orthodontic treatment.

Due to the extensive volume of the literature on automated cephalometric analysis, it is impractical to list all relevant studies. Therefore, this review focuses on summarizing pertinent literature, as detailed in Table 1, to highlight the past and the latest advancements in this field.

**Table 1:** Application of AI in orthodontics (past to present) AI: artificial intelligence, CNN: convolutional neural network, TMJ: temporomandibular joint, SS: spatial spectroscopy, BN: Bayesian network, ANN: artificial neural network.

S. No.	Author/Year	Study	Observation/Outcome
1	Levy et al., 1986 [8]	Knowledge-based landmarking of cephalograms	The study confirmed that the landmarking of cephalograms can be automated.
2	Rudolph et al., 1998 [9]	Automatic computerized radiographic identification of cephalometric landmarks	The findings revealed no statistically significant difference in the mean landmark identification mistakes made by automatic identification using spatial spectroscopy (SS) and manual identification on the computer display. Hence, spatial spectroscopy demonstrated potential for automated landmark detection.
3	Brown et al., 1991 [10]	The initial use of a computer-controlled expert system in the treatment planning of class II division 1 malocclusion	According to the study, an expert system generated treatment plans that were noticeably more palatable to a panel of specialists than the treatments that were actually carried out, even with an extremely tiny proportion of non-prior permission cases.
4	Leonardi et al., 2009 [11]	A CNN-based AI system for the automatic location of cephalometric landmarks	For automatic landmark detection, a CNN-based system achieved a satisfactory degree of accuracy.
6	Lu et al., 2009 [12]	ANN-based model for predicting post-orthognathic surgery image	The artificial neural network (ANN)-based system showed increased prediction accuracy and dependability.
7	Mario et al., 2010 [13]	A paraconsistent artificial neural network (PANN) for analyzing the cephalometric variables for orthodontic diagnosis	The specialist's performance and the models were identical. According to the author's conclusion, it can serve as a backup resource while making orthodontic decisions.
8	Xie et al., 2010 [14]	ANN-based AI model for deciding if extractions are necessary prior to orthodontic treatment.	ANN has shown to be efficacious in ascertaining the optimal treatment strategy for malocclusion patients: extraction or non-extraction.
9	Jung et al., 2016 [15]	Artificial intelligence expert system for orthodontic decision-making of required permanent tooth extraction	When comparing the system's suggestions for extraction with non-extraction, the models' success rates were 92%. Orthodontics may benefit from AI expert systems utilizing neural network machine learning.
10	Arik et al., 2017 [16]	AI-based deep (CNNs) for automated quantitative cephalometry	When compared to the best benchmarks in the literature, this system performed better.
11	Thanathornwong, 2018 [17]	Bayesian network (BN) for predicting the need for orthodontic treatment	When determining if orthodontic treatment is necessary, this BN-based method showed encouraging results with a high degree of accuracy.
12	Patcas et al., 2019 [18]	An AI system for describing the impact of orthognathic treatments on facial attractiveness and age appearance.	When evaluating patients undergoing orthognathic treatment, this CNN-based AI system can be utilized to score factors such as apparent age and facial beauty.
13	Patcas et al., 2019 [19]	An AI system for evaluating the facial attractiveness of patients who have undergone treatment for clefts and the facial attractiveness of controls and to compare these results with panel ratings performed by laypeople, orthodontists, and oral surgeons.	The results of the AI system for the cleft patients were similar to those of the other groups, while the controls had lower scores. It is necessary to further enhance this AI-based system.
14	Choi et al., 2019 [20]	ANN-based model for deciding on surgery/non-surgery and determining extractions	This artificial neural network (ANN) model showed a better success rate in selecting between surgery and non-surgery, as well as effective extraction decisions. Orthognathic surgery cases will benefit from the adoption of this ANN-based model for diagnosis.
15	Kok et al., 2019 [21]	AI algorithms for determining the stages of the growth and development of cervical vertebrae.	Cervical vertebral phases could be best determined using ANN.
16	Li et al., 2019 [22]	An ANN-based model for orthodontic treatment planning	The artificial neural network (ANN) system shown remarkable precision in forecasting extraction-nonextraction and extraction anchorage patterns. In order to help less experienced orthodontists anticipate orthodontic treatment, it can be helpful.
17	Makaremi et al., 2019 [23]	A CNN-based AI system for determining the degree of maturation of the cervical vertebrae.	Orthodontists can utilize this suggested model, which has been validated using cross-validation. Orthodontists can easily use this approved software.
18	Park et al., 2019 [24]	Comparing the latest deep-CNN-based systems for identifying cephalometric landmarks	The You-Only-Look-Once (YOLOv3) model was better than the Shot Multibox Detector in terms of accuracy and computing time.
19	Kunz et al., 2020 [25]	An automated cephalometric x-ray analysis using a specialized (AI) algorithm.	Similar to the competence level of skilled human examiners, an AI program was able to analyze unknown cephalometric X-rays.
20	Hwang et al., 2020 [26]	Deep-learning-based automated system for detecting the patterns of 80 cephalometric landmarks.	The accuracy of this system in recognizing cephalometric landmarks is comparable to those identified by human examiners. When repeatedly identifying different cephalometric landmarks, this approach could be a good choice.
21	Li et al., 2022 [27]	Temporomandibular joint segmentation in MRI images using deep learning	This study showed how CNN can recognize the articular disc automatically by segmenting the TMJ components.

Search strategy

An electronic search was conducted on PubMed, Web of Science, the Cochrane Central Library, and Google Scholar to find the relevant literature. The search was limited to English publications, and no filters were applied. Various combinations of the following keywords were used in the search procedure: artificial intelligence, machine learning, deep learning, neural networks, ANN, and orthodontics. No restrictions on the year or status of publications or languages were applied.

Applications of AI in orthodontics

Image Analysis and Diagnosis

Orthodontic diagnosis is challenging, as it requires a comprehensive assessment of various facial components from multiple angles. Digital dentistry now enables us to collect patient information digitally and store it in a database for diagnosis and treatment planning. Automation solutions utilizing AI and machine learning have significantly reduced the evaluation burden and minimized diagnostic variations.

Deep learning algorithms have been employed in the analysis of medical images, including those used in orthodontics. This includes the interpretation of X-rays, CT scans, and intraoral scans for orthodontic diagnosis and treatment planning. A thorough orthodontic diagnosis entails several evaluations, such as examinations of upper airway obstruction, skeletal maturation, facial anatomy, functional occlusion, and cranial anatomy. These evaluations assess the patient's skeletal maturity, facial features, dental and skeletal relation, and upper-airway patency in distinct ways.

An essential aspect of orthodontic treatment planning, outcome assessment, and diagnosis involves cephalometric analysis, particularly the identification of landmarks on lateral cephalograms. The precision and efficiency of clinical practice are impacted by manual landmarking since it takes time, depends on expertise, and can be inconsistent [28-31]. Even in the mid-1980s, automated landmark detection was attempted, but the early error margins were too large for practical use [32]. Recent advancements in AI have led to several studies focusing on enhancing the precision, effectiveness, and consistency of cephalometric analysis. Among the diverse uses of AI in orthodontics, cephalometric analysis has been extensively researched.

A total of 16 studies were included in the analysis of orthodontic data for diagnosis, problem classification, and orthodontic assessments. Similar to cephalometric landmark detection, most studies utilized deep learning techniques and artificial neural networks (ANN). Given the substantial amount of literature available, this review primarily aims to summarize relevant papers from the previous years, as outlined in Table 2, to present the recent developments in automated cephalometric analysis.

**Table 2:** Evidence-based applications for image analysis and diagnosis CNN: convolutional neural network, AI: artificial intelligence, ICC: intraclass correlation coefficient.

Sr. no.	Author/year	Study	Performance
1	Payer et al., 2019 [33]	Integrating spatial configuration into heatmap regression-based CNNs for landmark localization.	Error radii: 26.67% (2 mm), 21.24% (2.5 mm), 16.76% (3 mm), and 10.25% (4 mm).
2	Ryu et al., 2022 [34]	Classification of clinical orthodontic photos using deep learning.	According to this study, digital color pictures may be processed using artificial intelligence to help automate the orthodontic diagnostic procedure.
3	Li et al., 2022 [35]	Artificial Intelligence for classifying and archiving orthodontic images.	The DeepID-based modified model employed in this investigation proved to be quite good at categorizing orthodontic pictures. Additionally, deep learning can lessen the strain on dentists by improving the efficiency of dental follow-up and treatment.
4	Woo et al., 2023 [36]	Evaluating the accuracy of automated orthodontic digital setup models.	The software, tooth type, and movement dimension all affect how successful automated digital setup systems are. There has been a noticeable improvement in time efficiency.
5	Nishimoto et al., 2019 [37]	Personal Computer-Based Cephalometric Landmark Detection With Deep Learning, Using Cephalograms on the Internet.	Average prediction errors: 17.02 pixels. Median prediction errors: 16.22 pixels.
6	Moon et al., 2020 [38]	How much deep learning is enough for automatic identification to be reliable?	The accuracy of AI increases with a higher number of training datasets but decreases with an increase in the number of detection targets.
7	Hwang et al., 2020 [39]	Deep-learning-based automated system for detecting the patterns of 80 cephalometric landmarks.	The accuracy of this system in recognizing cephalometric landmarks is comparable to those identified by human examiners. When repeatedly identifying different cephalometric landmarks, this approach could be a good choice.
8	Zeng et al., 2021 [40]	Convolutional cascaded networks for automated detection of cephalometric landmarks.	The proposed approach attained the lowest mean radial error (MRE) and the highest success detection rate (SDR) for a precision range of 2.0 mm, which is clinically accepted, as well as for 2.5 mm, 3.0 mm, and 4.0 mm ranges.
9	Bulatova et al., 2021 [41]	Evaluation of automated identification of cephalometric landmarks using artificial intelligence.	There was no statistical difference for 12 out of 16 points when analyzing absolute differences between MT (manually traced) and AI groups.
10	Hong et al., 2022 [42]	Accuracy of landmark identification in serial lateral cephalograms of Class III patients who received orthodontic treatment and two-jaw orthognathic surgery with artificial intelligence assistance.	The total mean error was 1.17 mm, with no significant difference among the four time points (T0, 1.20 mm; T1, 1.14 mm; T2, 1.18 mm; T3, 1.15 mm). When comparing two time points ([T0, T1] vs. [T2, T3]), ANS, A point, and B point showed an increase in error (p < 0.01, 0.05, 0.01, respectively), while Mx6D and Md6D showed a decrease in error (all p < 0.01). There was no difference in errors at B point, Pogonion, Menton, Md1C, and Md1R between the genioplasty and non-genioplasty groups.
11	Le et al., 2022 [43]	Efficacy of human-artificial intelligence collaboration in detecting cephalometric landmarks.	The collaboration between beginners and AI improved the SDR by 5.33% within a 2 mm threshold and enhanced the success classification rate (SCR) by 8.38% compared to beginners alone. These findings indicate that the DACFL model is suitable for clinical orthodontic diagnosis.
12	Mahto et al., 2022 [44]	Assessment of fully automated cephalometric measurements obtained from a web-based platform driven by artificial intelligence.	All measurements demonstrated an ICC value exceeding 0.75. Seven parameters, including ANB, FMA, IMPA/L1 to MP (°), LL to E-line, L1 to NB (mm), L1 to NB (°), S-N to Go-Gn, achieved a higher ICC value (>0.9). Five parameters, including UL to E-line, U1 to NA (mm), SNA, SNB, U1 to NA (°), showed an ICC value between 0.75 and 0.90.
13	Uğurlu et al., 2022 [45]	Evaluation of an artificial intelligence algorithm based on Convolutional Neural Networks for automatic detection of cephalometric landmarks.	The CranioCatch AI system demonstrated high success detection rates for 21 anatomic landmarks in lateral cephalometric radiographs, with the sella point achieving the highest scores and the Gonion point the lowest, along with corresponding MRE values.
14	Duran et al., 2023 [46]	Assessment of the precision of fully automated cephalometric analysis software utilizing an artificial intelligence algorithm.	The consistency test revealed a statistically significant good level of consistency between Dolphin and OrthoDx™ measurements, as well as between Dolphin and WebCeph measurements, for angular measurements. However, a weak level of consistency was found for linear measurement and soft tissue parameters in both software.
15	Ye et al., 2023 [47]	Does automatic cephalometric software utilizing artificial intelligence outperform orthodontic experts in landmark identification?	Experimental results demonstrated that all three methods achieved detection rates exceeding 85% using the 2 mm precision threshold, considered acceptable in clinical practice. The Angelalign group achieved a detection rate exceeding 78.08% using the 1.0 mm threshold. The AI-assisted group exhibited a significant difference in time compared to the manual group due to heterogeneity in landmark detection performance.
16	Bao et al., 2023 [48]	Assessing the precision of automated cephalometric analysis utilizing artificial intelligence.	The automatic program achieved an MRE of 2.07 ± 1.35 mm for 19 cephalometric landmarks, with soft tissue landmarks (1.54 ± 0.85 mm) showing greater consistency than dental landmarks (2.37 ± 1.55 mm). Overall, 15 out of 23 measurements were within the clinically acceptable level of accuracy (2 mm or 2°), and consistency rates within the 95% limits of agreement were all above 90% for all measurement parameters.

AI has demonstrated effectiveness in diagnosing and assessing orthodontic problems. Orthodontic treatments for malocclusion are generally categorized as extraction or non-extraction treatments. The choice between these methods is traditionally guided by clinical expertise, which can be challenging for inexperienced practitioners. Deep learning techniques have been suggested as a solution to assist in making such decisions [49].

Assessing the necessity for orthognathic surgery can be intricate. AI has exhibited the potential to aid clinicians in determining the need for surgical intervention [43]. Cephalometric analysis plays a crucial role in diagnosing facial growth irregularities in orthodontics. Manual identification of cephalometric landmarks on X-ray images is laborious and error-prone. Recent research has shown notable advancements in landmark detection through AI techniques, especially deep learning models [39]. Additionally, AI now enables researchers to forecast post-orthognathic surgery aesthetics, providing a critical factor for surgical decision-making [50].

Automated Treatment Planning

Deep learning has shown promise in automated diagnosis and treatment planning. Algorithms can analyze patient data and propose treatment plans based on established orthodontic principles. Thorough decision-making is necessary while undergoing orthodontic treatment, especially when deciding on important details like the extraction strategy and whether or not surgery will be required. It is expected that AI will help orthodontists, especially inexperienced ones, make wise selections.

Tooth crowding and protrusions are primary reasons for tooth extraction in orthodontics. While there is no definitive guideline for extractions, experienced specialists' decisions can guide less experienced practitioners. Studies have identified orthodontic features using cephalometric data, patient photographs, and clinical examinations, mostly utilizing artificial neural networks (ANN). Xie et al. used an ANN to detect the need for tooth extraction, focusing on anterior teeth not covered by lips and the incisor mandibular plane angle (IMPA), though their study had limited generalizability [14]. Jung and Kim addressed extraction positions, using validation and test sets to prevent overfitting, but their study was limited to nonsurgical cases [15].

Choi et al. achieved a 91% success rate in diagnosing extraction needs in orthognathic surgeries [20]. Li et al. reached 94% accuracy in extraction decisions, outperforming Xie et al. by 14%, using a more stable feature importance method [27, 14]. Suhail et al. employed a random forest algorithm but excluded certain extractions and focused on nonsurgical procedures [51].

Table 3 provides an overview of how AI is used in treatment planning. In the reviewed studies, machine learning and deep learning techniques were employed for decision-making in orthodontic treatment planning. These included determining whether to extract teeth (with accuracies ranging from 80% to 96%), evaluating treatment procedures, and deciding when to terminate orthodontic treatment (with accuracies between 94.2% and 98.7%). The techniques were also used to choose between orthognathic surgery and orthodontic treatment (with accuracies between 91.9% and 96%), predict blood loss before orthognathic surgery (showing a high correlation between predicted and actual blood loss values, with P = 0.001), design treatment plans with free-form certificates (F1 score of 58.5), and identify key features contributing to the success of pre-orthodontic treatments for cleft lip and palate patients (with an accuracy of 88.89%) [50].

**Table 3:** Evidenced-based applications for automated treatment planning AI: artificial intelligence, ML: machine learning

S. No	Author/Year	Study	Observation/Outcome
1	Suhail et al., 2020 [51]	Machine learning for the diagnosing of orthodontic extractions	The machine learning method was able to anticipate the extraction process with an accuracy that is about equivalent to that achieved by many experts despite the small feature set.
2	Etemad et al., 2021 [52]	Machine learning from clinical data sets of a contemporary decision for orthodontic tooth extraction	This work progressed the field's research by extending its application to a broader population in the United States, which possesses the largest patient pool and the greatest number of clinical variables among the four models tested. We discovered that a minority special sample existed in our data set that cannot be reliably predicted using the current classic algorithms. To use machine learning models in the orthodontic field, more performance improvement is required.
3	Shojaei et al. 2022 [53]	Constructing machine learning models for orthodontic treatment planning	The outcomes show that a medical diagnostic model using a neural network may produce a high degree of accuracy.
4	Real et al., 2022 [54]	Use of AI to predict the need for orthodontic extractions	Using an AutoML technique, three separate models were created and evaluated to predict the orthodontic need for dental extractions. The models achieved accuracy rates of up to 93.9% for tooth extraction predictions, which was comparable to results from more sophisticated methods.
5	Leavitt et al., 2023 [55]	To investigate the utility of machine learning in predicting orthodontic extraction patterns	The most predictive indicators for determining extraction patterns were found to be the molar relationship, mandibular crowding, and overjet. All of the tested supervised machine learning techniques produced good accuracy in predicting the U/L4s and U4s extraction patterns, but they predicted poorly for the U4/L5s, U5/L4s, and U/L5s extraction patterns.
6	Prasad et al., 2023 [56]	ML predictive model as clinical decision support system in orthodontic treatment planning	In comparison to the treatment plan determined by orthodontists with expert opinion for identical patients, the ML-based AI model demonstrated 84% accuracy in predicting the treatment plan. Also, it forecasted how much each diagnostic data set will contribute to the choice of treatment strategy.

Facial Recognition and Analysis

Deep learning models have been used for facial recognition and analysis, aiding orthodontists in understanding the facial structure and predicting the effects of orthodontic interventions on a patient's appearance. Orthognathic surgery, when combined with orthodontic therapy, is frequently required to realign the jaws in adults with substantial dentofacial anomalies. However, there are no clear-cut standards for determining whether surgical intervention is necessary, which can be confusing for novice orthodontists, particularly in circumstances where the choice between surgery and camouflage orthodontic treatment is unclear [57-59].

Lateral cephalograms are frequently employed in clinical settings to assess sagittal skeletal anomalies. Achieving accuracy rates above 90%, several studies have utilized lateral cephalograms as input data for convolutional neural networks (CNNs) or artificial neural networks (ANNs) [60, 61]. In their training dataset, Shin et al. incorporated both lateral and posteroanterior (PA) cephalograms to evaluate the sagittal as well as lateral relationships of the jaws. Their CNN model achieved a 95.4% accuracy in predicting the need for orthognathic surgery [61].

Another important factor in deciding between surgery and non-surgery is facial attractiveness. Knoops et al. utilized support vector machines (SVMs) to accurately predict, with a 95.4% success rate, the necessity of surgery based on 3D face scans [62]. Conversely, Jeong et al. trained a CNN model on front and right facial photos but achieved a lower accuracy of 89% [63]. Choi et al. employed a variety of factors, including cephalometric measurements, dental characteristics, profile attributes, and the complaint associated with forwardly placed teeth, as part of the training data. The proposed ANN model demonstrated an accuracy rate between 88% and 97%, successfully predicting both the need for surgery and the decision to extract teeth in surgical scenarios. However, this study did not include Class I surgical cases, potentially affecting the generalizability of the model to wider populations [20]. Lee et al. explored the predictive capabilities of random forests (RF) and logistic regression (LR) in ascertaining the necessity for surgery in Class III subjects, achieving accuracies of 90% (RF) and 78% (LR) with comparable input data [60]. 

Even though artificial intelligence (AI) has made strides in orthognathic surgery decision-making, further development is required to cover a wider variety of cases, particularly borderline cases, in order to improve AI's diagnostic skills. The application of AI in facial analysis is depicted in Table 4.

**Table 4:** Evidence-based applications for facial recognition and analysis AI: artificial intelligence.

S. No.	Author/Year	Study	Observation/Outcome
1	Tanikawa et al., 2021 [64]	Machine learning for facial recognition	The study aimed to develop AI systems predicting post-treatment facial morphology after orthognathic surgery and orthodontic treatment, challenging the assumption of proportional movement between hard and soft tissues in current algorithms, which lack evidence for their prediction validity.
2	Rousseau and Retrouvey, 2022 [65]	Automated facial analysis of vertical dimension for increased precision and efficiency	The study utilized an automated approach for soft-tissue measurements, which offers enhanced reliability and efficiency compared to manual digital methods, presenting a valid alternative. This method, supported by machine learning, can aid orthodontic clinicians and researchers in advancing evidence-based practice, particularly in studying longitudinal changes in the vertical dimension and clinical diagnosis.

Predictive Modeling

Predictive modeling using deep learning techniques helps in forecasting the outcomes of orthodontic treatments. This includes predicting tooth movement and assessing the impact of different treatment options. Orthodontists often develop multiple treatment plans, especially for borderline cases, which can be challenging to decide on, particularly for inexperienced practitioners. Unsatisfactory treatment outcomes and permanent consequences might result from suboptimal plans, especially those that include extraction and interproximal enamel loss. In order to reduce potential dangers and complications, orthodontists can analyze and treat malocclusions more scientifically with the use of treatment outcome prediction. AI can currently aid in predicting dental, skeletal, and facial changes, as well as patient experiences with clear aligners, thereby guiding treatment planning [66, 67].

Although Kesling originally suggested orthodontic tooth setup, which involves labor-intensive manual procedures such as tooth segmentation and repositioning, it allows for the visualization of treatment progress and ultimate occlusion. With ongoing advancements in digital orthodontics and AI, automated virtual setups are increasingly utilized, particularly in the realm of clear aligners. In a study by Woo et al., the six directions of tooth movement were compared between the accuracy of three automated digital-setting software and human setup. Although the study found that the automatic virtual setup software was usually effective, further human modifications could still be required in clinical practice. Crucially, the analysis only comprised instances in which no extractions were carried out [36].

Several studies have examined the application of AI to predict changes to the skeletal and facial structures after orthodontic treatment, in addition to changes to the teeth. A study conducted by Park et al. involved the utilization of modified C-palatal plates on Class II individuals. The study employed a Convolutional Neural Network (CNN) model to predict cephalometric changes, demonstrating an accuracy of 1.79 ± 1.77 mm [66]. In a separate study, Tanikawa et al. combined geometric morphometric techniques with deep learning to anticipate 3D facial changes following orthodontic treatment and orthognathic surgery. The surgical and orthodontic groups achieved average error values of 0.94 ± 0.43 mm and 0.69 ± 0.28 mm, respectively [67]. Additionally, a study was conducted to determine the impact of incisor movement, age, and gender on 3D facial alterations following orthodontic treatment. A conditional generative adversarial network (cGAN) was utilized to achieve an accuracy rate of 97.5% with a mean prediction error of 1.2 ± 1.01 mm [68].

Choosing the right treatment appliance is essential when planning orthodontic treatment, particularly for patients undergoing clear aligner therapy, as a negative wearing experience can impact patient adherence and treatment results. Xu et al. utilized the training data of 17 subjects to develop an artificial neural network (ANN) model that could predict the experiences of patients undergoing Invisalign treatment. Their model achieved a prediction accuracy of 92% for quality of life, 93% for anxiety, and 87% for pain [69]. This work lays the groundwork for future research in this field by being the first and, as of now, the only one to use AI to predict patient experiences with orthodontic treatment. One drawback of this research is that it only used the clinical characteristics of the patients as input data; it ignored other variables that can have an impact, such as gender and educational attainment, which could affect the model's predictive capacity.

Computer-Based Decision Support System

Treatment planning is a critical phase in the orthodontic procedure. AI enhances this process by incorporating knowledge gained from experienced experts. Predicting treatment outcomes assists clinicians in making more informed decisions. Anticipating aesthetic and clinical results can help both the surgeon and the patient make the best possible choices.

The deep learning algorithm 3D U-Net is mostly used for 3D image segmentation. A modified version, 3D-UnetSE, incorporates squeeze-and-excitation modules, demonstrating superior performance in capturing high-level characteristics. Palatal micro-implant stability is impacted by both soft and hard tissues. The team led by Tao et al. utilized a 3D-UnetSE tool to determine optimal locations for palatal miniscrews. This was achieved through the automated evaluation of hard palate and soft tissue thickness using CBCT. By doing so, the team was able to make informed decisions on the best locations based on the thickness of the bone and soft tissues [70].

Monitoring tooth root locations during orthodontic treatment is essential to preventing unfavorable outcomes and evaluating treatment success. Traditional modalities such as cone beam computed tomography (CBCT) or panoramic radiography contribute to elevated radiation exposure. Hu et al. and Lee et al. used deep learning to create integrated tooth models. They integrated intraorally scanned dental crowns with precisely segmented teeth using CBCT images. Orthodontists may now identify the position of the tooth root using intraoral scanning. These studies showcase the promising ability to predict tooth positions, with continual enhancements anticipated to expand the use of integrated models in clinical settings [29].

Deep learning has been integrated into clinical decision support systems to assist orthodontists in making informed decisions about treatment plans. These systems can provide recommendations based on a large dataset of patient cases.

Radiographs and clinical images are often used in orthodontics for monitoring therapy and diagnosis. Using AI to assist in the classification and categorization of these images can improve the efficacy of healthcare practice. Ryu et al. employed convolutional neural networks (CNNs) to automatically classify intraoral and facial photos, yielding an overall correct prediction rate of 98% [34]. Li et al. increased the number of orthodontic picture categories to 14 by using a deep learning model based on Deep Hidden IDentity (DeepID). In addition to one panoramic video and one lateral cephalogram, these pictures featured six distinct facial and six intraoral photographs. With the use of joint Bayesian for verification and deep convolutional networks for feature extraction, the DeepID model obtained a high accuracy of 99.4% while also greatly accelerating computation [35].

Patient Monitoring

Deep learning models have been applied to monitor the progress of orthodontic treatment. This involves sequential images or scans to assess changes in tooth alignment and overall treatment effectiveness. Orthodontists can monitor treatment progress and offer feedback using photographs or oral scans, reducing unnecessary frequent visits for patients [71, 72].

AI has improved the functionality and efficacy of remote monitoring software [71]. One prominent example of AI-driven remote monitoring software that is gaining traction and academic interest is dental monitoring (DM) [72]. With DM, patients may conveniently capture images of their teeth using a smartphone. Research indicates that DM enhances patient compliance while also cutting down on chairside time [71]. It is compatible with transparent aligners and fixed appliances, and it can instantly identify problems including misfitting aligners, missing attachments, bracket breakage, and relapse [71]. Furthermore, DM's detections are quite accurate. According to Homsi et al., intraoral scans and digital models produced by DM were equally precise [73]. Comparable precision was observed by Moylan et al. when measuring the intercanine and intermolar widths [74]. However, the extensive use of AI-driven remote monitoring tools should be done with caution. A recent study found inconsistent DM recommendations, especially in relation to teeth that have tracking problems and obvious aligner replacement.

Limitations and future

Recent advancements in artificial intelligence (AI) have significantly impacted orthodontics, particularly in clinical practice, treatment planning, and diagnosis. This comprehensive review provides a detailed overview of the latest AI developments within the field. While these advancements hold promise for improving orthodontic care, several challenges hinder their widespread adoption.

The reliability of current research is compromised by the limited and non-representative training data availability. Although certain AI models demonstrate high accuracy, their ability to perform well on uncommon deformities is questionable due to inadequate representation in the training dataset. Approaches like data augmentation, semi-supervised learning, and few-shot learning are intended to mitigate data scarcity, but their efficacy is constrained [75]. To minimize the requirement for a large amount of training data, transfer learning uses pre-trained models in similar domains; however, these models may not be as generalized when applied to other domains [76]. Data augmentation can increase sample size but cannot improve biological variability [75]. Semi-supervised learning still requires a large amount of unannotated data and high-quality annotations even with limited annotated data [75]. Few-shot learning faces challenges due to the absence of standardized assessment frameworks and specialized data [77]. Ethical and privacy considerations make data sharing difficult, resulting in biased AI models trained on data with limited generalizability [78]. Potential solutions to enable data sharing while maintaining data security and privacy include federated learning and blockchain technology, which can result in larger and more varied datasets [79, 80].

Moreover, variations in research methodologies, dataset magnitudes, and evaluation criteria pose challenges in comparing different studies. The minimum-information-about-clinical-artificial-intelligence modeling (MI-CLAIM) framework, proposed by Norgeot et al., seeks to resolve this by establishing uniform standards for effectiveness and transparency in clinical AI modeling [81]. Furthermore, despite the remarkable capabilities of AI algorithms, especially deep learning, there are ongoing concerns regarding their interpretability. Explainable AI (XAI) techniques aim to improve the clarity and understandability of AI algorithms. Approaches like gradient-weighted class activation mapping (Grad-CAM) and DeConvNet can uncover the underlying features influencing decision-making, which could improve the interpretability of AI models in orthodontics [82].

Finally, overfitting is a prevalent problem in AI, characterized by models performing well on training data but poorly on testing data. Techniques such as enhancing data samples, data augmentation, cross-validation, and selecting appropriate algorithms can mitigate overfitting [83]. However, not all studies in this review have addressed overfitting concerns. Despite these hurdles, AI holds significant promise in orthodontics, particularly in automating the identification of orthodontic treatment requirements and managing treatment complexities. With the continuous expansion of clinical data availability and AI computational capabilities, significant advancements in orthodontics are anticipated [84, 85].

## Conclusions

AI shows promise as a valuable aid in conducting diagnostic assessments, prognostic predictions, and treatment planning, particularly for complex cases. However, current scoping review results suggest that widespread clinical use of end-to-end machine learning tools is still a distant goal. The most promising applications of AI in orthodontics include landmark detection on lateral cephalograms, skeletal classification, and decision-making regarding tooth extraction.

In conclusion, the integration of deep learning in orthodontics represents a transformative leap toward more accurate, personalized, and efficient patient care. The burgeoning field has witnessed substantial progress in image analysis, diagnosis, treatment planning, and research, offering numerous advantages while acknowledging certain challenges. As we anticipate future developments in this dynamic field, it is imperative to prioritize ongoing research, ethical considerations, and the establishment of robust regulatory frameworks. By doing so, the integration of deep learning in orthodontics is poised to redefine the standard of care, ushering in an era of precision medicine and data-driven decision-making in orthodontic practice.
